# Air Quality Strategies on Public Health and Health Equity in Europe—A Systematic Review

**DOI:** 10.3390/ijerph13121196

**Published:** 2016-12-02

**Authors:** Li Wang, Buqing Zhong, Sotiris Vardoulakis, Fengying Zhang, Eva Pilot, Yonghua Li, Linsheng Yang, Wuyi Wang, Thomas Krafft

**Affiliations:** 1 Faculty of Health, Medicine and Life Sciences, Maastricht University, Maastricht 6200 MD, The Netherlands; li.wang@maastrichtuniversity.nl (L.W.); eva.pilot@maastrichtuniversity.nl (E.P.); 2 State Key Laboratory of Environmental Criteria and Risk Assessment, Chinese Research Academy of Environmental Sciences, Beijing 100012, China; zhongbq@craes.org.cn; 3 Environmental Change Department, Centre for Radiation, Chemical and Environmental Hazards, Public Health England, London WC1E 7HT, UK; Sotiris.Vardoulakis@phe.gov.uk; 4 Department of Environmental Quality Comprehensive Assessment, China National Environmental Monitoring Center, Beijing 100012, China; zhangfy@cnemc.cn; 5 Key Laboratory of Land Surface Pattern and Simulation, Institute of Geographical Sciences and Natural Resources Research, Chinese Academy of Sciences, Beijing 100101, China; yhli@igsnrr.ac.cn (Y.L.); yangls@igsnrr.ac.cn (L.Y.); wangwy@igsnrr.ac.cn (W.W.)

**Keywords:** air quality, strategy, assessment, health, health equity, systematic review

## Abstract

Air pollution is an important public health problem in Europe and there is evidence that it exacerbates health inequities. This calls for effective strategies and targeted interventions. In this study, we conducted a systematic review to evaluate the effectiveness of strategies relating to air pollution control on public health and health equity in Europe. Three databases, Web of Science, PubMed, and Trials Register of Promoting Health Interventions (TRoPHI), were searched for scientific publications investigating the effectiveness of strategies on outdoor air pollution control, public health and health equity in Europe from 1995 to 2015. A total of 15 scientific papers were included in the review after screening 1626 articles. Four groups of strategy types, namely, general regulations on air quality control, road traffic related emission control interventions, energy generation related emission control interventions and greenhouse gas emission control interventions for climate change mitigation were identified. All of the strategies reviewed reported some improvement in air quality and subsequently in public health. The reduction of the air pollutant concentrations and the reported subsequent health benefits were more significant within the geographic areas affected by traffic related interventions. Among the various traffic related interventions, low emission zones appeared to be more effective in reducing ambient nitrogen dioxide (NO_2_) and particulate matter levels. Only few studies considered implications for health equity, three out of 15, and no consistent results were found indicating that these strategies could reduce health inequity associated with air pollution. Particulate matter (particularly fine particulate matter) and NO_2_ were the dominant outdoor air pollutants examined in the studies in Europe in recent years. Health benefits were gained either as a direct, intended objective or as a co-benefit from all of the strategies examined, but no consistent impact on health equity from the strategies was found. The strategy types aiming to control air pollution in Europe and the health impact assessment methodology were also discussed in this review.

## 1. Introduction

Despite efforts to control and reduce air pollution in many countries, ambient (outdoor) air pollution in both urban and rural areas was estimated to have been associated with up to 3.7 million premature deaths worldwide in 2012, with a significant proportion of these deaths in Asia (mainly in China and India) [1]. Air pollution has been associated with multiple diseases, such as cardiovascular diseases, asthma exacerbations, lung cancer, and diminished life expectancy [2,3,4,5,6]. Further, these negative health impacts varied according to the socioeconomic position (SEP) and health condition of individuals [1]. Research findings showed that people with disadvantaged social-economic status were more likely to be exposed to higher air pollutant concentrations in ambient environments, at home, in school, in occupational environments, in the neighbourhood, and in commuting [7,8,9,10,11,12,13]. Epidemiological studies also showed that specific population groups, such as the elderly, young children and people with pre-existing respiratory or cardiovascular conditions, are more likely to be affected by air pollutants, indicating that air pollution could increase health inequity [11,14,15].

Air pollution control efforts in Europe extend to more than a century. Some early examples are the Alkali, &c. Works Regulation Act (1906) and the Clean Air Act (1956) following the “Great London Smog” of 1952 in the UK [16,17]. Since then, a series of pieces of legislation and programmes have been put forward, such as the European Union (EU) Directives which set limit values and guidelines for air pollutants [18,19]; the Convention on Long-range Trans-boundary Air Pollution (CLRTAP) and related protocols, which focused on emission reductions for specific air pollutants [20]; the Clean Air for Europe Programme (CAFE 2005) which facilitated the establishment of air pollution control strategies to protect human health [21]; and local actions such as low emission zones (LEZs) and the introduction of vehicle exhausts catalysts (VECs) to control traffic emissions [22]. With these legislations and programmes, the air quality in EU has improved in recent decades, particularly for the Western EU member countries. However, in more than one third of EU’s Air Quality Zones, particulate matter (PM) concentrations exceed the limit values, and the limit values for nitrogen dioxide (NO_2_) are not met in about a quarter of the zones [23]. As a consequence, a large proportion of the urban population, particularly those living close to heavily trafficked roads or industries, and those living in large city centres, remain exposed to air pollutants with concentrations that exceed the European air quality standards for outdoor air quality. More specifically, in 2011, 33% of the urban population in the EU-27 were exposed to PM_10_ levels (particles with aerodynamic diameter less than 10 micrometres) exceeding the daily limit value; 31% were exposed to PM_2.5_ levels (particles with aerodynamic diameter less than 2.5 micrometres) exceeding the annual exposure concentration obligation; still 5% were exposed to NO_2_ concentrations exceeding the annual limit value; and 14% were exposed to higher ozone concentration than set in the EU target value [8,23,24]. It is estimated that in 2010, 406,000 premature deaths were attributable to exposure to particulate matter and ground-level ozone in Europe [23]. PM_2.5_ would be still responsible for 5.5 months statistical loss of life on average across the EU by 2020 and OECD countries are likely to have one of the highest rates of premature death from ground-level ozone by 2050 if there are no more stringent strategies to control air pollution [21,25]. Although an increasing number of strategies have already been introduced from EU level to local level [18,19,21,26,27,28], and several epidemiological studies on air pollution and its adverse health impact have been carried out [8,29,30,31], there is no comprehensive summary of the effectiveness of air pollution control strategies on public health , and particularly on health equity in the EU.

In order to review the effectiveness of air pollution control strategies and to understand their impacts on public health and health equity, we undertook a systematic review of relevant published studies focusing either on health impact assessment or on health equity assessment in Europe. This review aimed to examine health equity associated with air pollution control strategies, to provide scientific suggestions for further studies on air pollution control strategies, and importantly, to provide evidence on the effectiveness of air pollution control strategies in Europe, which may be transferable to Asian countries where air pollution is posing a very significant public health challenge.

## 2. Methods

### 2.1. Definition and Searching Method

Summarized from WHO glossary of terms used for Health Impact Assessment (HIA), health inequities were defined as uneven health status which may be unnecessary and avoidable as well as unjust and unfair, and these differences in health status are attributable to the external environment and conditions mainly outside the control of the individuals concerned. Health inequalities were differences in health status or in the distribution of health determinants between different population groups, and those differences are attributable to biological variations, free choice, or the external environment and conditions mainly outside the control of the individuals concerned (this also applies to health inequity) [32]. There are differences in health outcomes from exposure to air pollution, which may affect more socioeconomically deprived groups that often live near busy roads or industrial sites and have fewer opportunities to move to less polluted and usually more expensive areas [9,11,12]. Furthermore, air pollution disproportionately affects the more susceptible groups, including the elderly and those with pre-existing illness. In this review, we summarized the strategies on air quality control and their impacts on health and health equity. The objective of this study was to explore the effectiveness of the air pollution control related strategies on public health and health equity, and the strategies in this review were limited to: (1) specific strategies such as policies, regulations, legislation, or directives on ambient air quality control at EU or national level; and (2) specific interventions or actions to reduce ambient air pollution emissions.

We conducted a systematic literature review aiming to assess public health and health equity impact of air quality control strategies based on three databases, Web of Science, PubMed, and Trials Register of Promoting Health Interventions (TRoPHI). In Web of Science, we searched on the scope, which includes title and abstract; in PubMed, we searched on the title and abstract; and in TRoPHI, on keywords. Strategy in our review was defined as interventions, policies or directives aimed at reducing air pollutants or where concentration reduction occurred as an unintended consequence of a strategy. Four key themes, air quality, strategies, health and effectiveness, were selected, and search terms for each theme were selected and defined by consent of all authors. For air quality, we used the following search terms: “air pollution”, “air pollutant”, “outdoor air”, “ambient air”, and “atmospheric air”. For strategies, we used the following search terms, “policy”, “programme”, “project”, “regulation”, “management”, “plan”, “strategy”, “action”, “directive”, “intervention”, “emission control”, “scheme”, and “initiative (in the way of campaign, training, incentive, etc.)”. For health, we used the search terms of “health equity”, “health inequity”, “mortality”, “death”, “morbidity”, and “health”. For effectiveness, we included the following search terms, “evaluation”, “assessment”, “efficacy”, “effectiveness”, “efficiency”, and “impact”. Regarding health equity impact, for those studies with health equity assessment, we summarized the effectiveness of the strategies in improving health equity. For those studies without or with no direct assessment of health equity, we commented on the capacity of the strategies to influence health equity based on whether the studies mentioned health gains from the strategies in terms of differential air pollution reductions (geographical distribution), or different health response among different groups, including age groups, pre-existing health condition groups, gender groups and socioeconomic groups. Those publications containing at least one term of each theme were identified in this search. The inclusion and exclusion criteria are shown in Table 1.

### 2.2. Screening Method

For the period 1 January 1995 to 4 October 2015, 1626 articles were identified from the three databases according to the searching terms, 1522 from Web of Sciences (with country restriction), 103 from PubMed (refined by “Europe” in Mesh) and 1 from TRoPHI.

**Figure 1:**
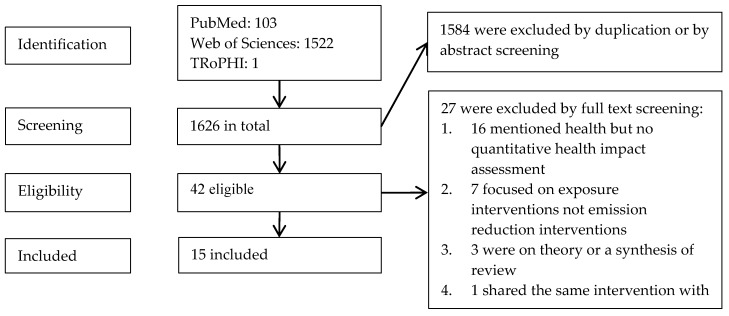
Flow chart of the searching and screening procedure.

Among these, 1584 were excluded after the duplication check in Endnote and abstract screening, and 42 selected for full text screening (Figure 1). From these 42, 16 articles were excluded because they mentioned health but with no quantitative health impact assessment; 7 articles were excluded because the strategies were not about air pollution source emission control but exposure interventions, such as improved ventilation system or decreased exposure frequency; 3 articles were excluded because the studies were on theory or a synthesis of existing reviews on air quality policies and health impact assessment; and 1 was excluded because it shared the same strategy and air pollution outcome with one included study [33,34]. At the end, 15 articles were included after the full text screening.

## 3. Results

Based on the study types of the 15 articles included, we divided the articles into three categories (marked with I, II and III, see Table 2). The first category comprised articles tackling air pollution under the scope of general guidelines at WHO, EU or national level, by means of policies, directives, legislation, standards, guidelines or targets (I); the second category comprised articles on specific interventions, by means of actions or experimental studies (II); and the third category covered articles assessing air quality changes and health benefits under different scenarios (III).

Articles under category I, covered larger geographic areas, for example the entire EU or member countries; while for those with specific actions, the area was smaller and more on the level of a selected city or even a smaller area. There were five articles from the United Kingdom (UK), three articles at European level, two articles from Spain, and one article each from Hungary, Sweden, German, Italy and Ireland. All articles focused on urban areas (13 exclusively, while two covered both urban and rural areas). In most of the articles, the target pollutants were particulate matter (PM_10_, PM_2.5_), followed by NO_x_ (nitrogen oxides), SO_2_ (sulphur dioxide) and ozone, and the pollutant concentrations were assessed by real-time monitoring, model simulation or were set as targeted concentrations according to guidelines or seniors.

In order to explore the impact of the strategies on public health and health equity, we only included articles with health outcome indicators. The health variables used in these articles can be classified into three categories. Most of the articles used attributable mortality (including premature mortality) and morbidity, including hospital admissions and some specific symptoms such as nasal, ocular and respiratory symptoms; some used different ways of relating the health outcome to life years, including life expectancy or years of life gained (YLG); and some used monetized health benefits.

For the health impact assessment, the exposure-response relationships were mostly derived from long-term or both short and long term exposure assessments and only four focused on short-term exposure effects. Most of the articles focused on the entire population, stratified by age group, by gender, by socioeconomic position, or by the distance to the intervention areas within the catchment of the respective study areas. Table 3 summarizes the health impact assessments and comments on health equity based on the type of the strategies.

### 3.1. Summary of the Strategy Types

As shown in Table 2, articles can be categorized into three different study types, I [33,39,48], II [35,36,37,38,40,43,44,46,47] and III [41,42,45], respectively. We also classified strategies and interventions into four categories (Table 3), namely focusing on general regulations on air quality control, energy efficiency or saving, transport related emission reductions, and greenhouse gas emission reductions. The review showed that, for the real time monitored air pollution studies, the air pollution concentrations or emissions decreased as the consequence of implementing the respective strategies. For the scenario simulation studies, health impact assessment was conducted on the premise that the air pollutants decrease to some extent after implementation of pollution control strategies. Although we have generalized the strategies into four categories, several of these strategies were crosscutting and could fall into several of these categories, for example, the intervention aiming to reduce greenhouse gas emission by introducing lower-carbon emission motor vehicles was also a traffic emission control related intervention [44].

**General regulations on air quality control:** For the articles concerning general regulations on air pollution control, the air pollution outcomes were targeted through regulations or guidelines (study type I). These general regulations included the UK National Air Quality Strategy, EU air quality Directives, WHO air quality guidelines, U.S. Environmental Protection Agency guidelines and Spain pollution control policies. All of the studies included focused on particulate matter, which indicates that particulate matter, particularly PM_2.5_, was one of the major concerns for public health associated with air pollution in Europe in recent years. This was reflected in the EU Directive 2008/50/EC on Ambient Air Quality and Clean Air for Europe which first set short and long term targets for PM_2.5_ [19]. Most of the actions aiming at decreasing air pollution were implemented to meet the targets set up by EU, while climate change mitigation interventions were based on the scenarios of IPCC’s Fifth Assessment Report (AR5) to limit global temperature increase to 2 °C by the end of this century [48]. However, greenhouse gas emission reductions can be achieved simultaneously with air pollutants emission reductions, since both are often emitted from the same sources, such as from the transport and energy sectors [35,44].

**Energy related strategies:** With regard to the energy related strategies, we analysed three studies aiming to reduce air pollution emissions [33,35,36], and one study in which the air pollution decrease was the co-benefit of a climate change mitigation intervention [44]. The strategies were the Energy Saving Program of Hungary’s National Energy Efficiency Improvement and Energy Conservation Programs [35], the ban of coal sales in Dublin, Ireland [36], and the European Commission Directives to reduce the sulphur content in liquid fuels for vehicles [33]. These strategies mainly focused on the reduction of SO_2_ concentrations in ambient air, followed by NO_x_, black smoke, and total suspended particles (TSP). The ban of coal sales obtained the most substantial SO_2_ reduction by 33.8% during the study period. While for the co-benefit strategies, carbon emission reduction was the main objective and outdoor particulate matter concentrations decrease accordingly as a by-product through the introduction of low carbon emission measures for motor vehicles.

**Traffic emission control related interventions:** Six articles focused on traffic related emission control. Among these, two focused on Low Emission Zones [46,47]; two were about congestion charging schemes for vehicles entering into specific areas [39,42]; one was about installing vehicle exhaust catalysts [38]; and one was an experimental study on constructing a by-pass road [37]. As a co-benefit from controlling traffic congestion intervention, introducing access restrictions to specific areas resulted in air pollutant reductions and health benefits [40,43]. The air pollutants targeted were mainly NO_x_ and particulate matter for traffic emission control interventions. Congestion Charging and Low Emission Zones were two typical methods in Europe aimed at either reducing traffic congestion or controlling traffic emissions from the most polluting vehicles in European cities, and both of them can reduce traffic related pollution emissions within the zones.

Congestion Charging was first introduced in Singapore in 1975, with the stated objective to reduce traffic congestion and traffic emissions. The Stockholm congestion tax system (similar with CCS) did decrease emissions of NO_x_ and particulate matter by up to 12% and 7% according to the test trail from 2003 to 2007 [43], and for the Congestion Charging Scheme in London, the average ambient NO_2_ and PM_10_ concentrations declined moderately within the zone [40].

LEZs are areas where only vehicles with pollutant emission levels lower than a defined limit are allowed to enter, or alternatively, access charges are taken from vehicles that have higher emission levels. More than 200 LEZs have already been implemented in Europe, mainly in major cities, such as Berlin, Amsterdam, London, Lisbon and Rome, aiming to reduce exhaust emission of particulate matter and NO_x_, and studies indicated those LEZs in general improved air quality, particularly in the vicinity of busy roads [49,50]. Cyrys et al. (2014) reviewed the impact of LEZs on air quality and health, and found that LEZs in Berlin did contribute to the traffic emission reductions, with 10% reduction in PM_10_ and 58% in diesel particle concentrations [47]. While for the LEZ study in Rome, using model simulation, Cesaroni et al. (2011) found reductions in NO_2_ (23%) and PM_10_ (10%) concentrations after the implementation of the LEZ, but the reductions were mainly in the intervention area not the whole city [46].

Vehicle Exhaust Catalysts (VECs), which are largely used in Europe, were introduced to reduce the NO_x_, VOCs and CO emissions from petrol fuelled vehicles. A study from the UK indicated that VECs provided substantial pollutant concentration reductions, 20% for NO_2_, 10% for PM_10_, 30% for VOCs and 70% for CO [38]. Aside from LEZs, VECs and Congestion Charging, in order to reduce traffic related emissions or in some cases to alleviate traffic congestion, European cities introduced some other traffic control strategies, such as Traffic Limited Zones and Traffic Restrictions [22]. Although the traffic related interventions included in this study reported moderate to significant improvement in air quality, other studies showed less consistent results. The Ecopass zone in Milan (Traffic Restriction) led only to minor reductions of PM_10_ and PM_2.5_ concentrations but to a significant reduction of black carbon in a three-day experimental study [51]; similarly, non-significant reductions in NO_2_, NO_x_ and soot were observed in five Dutch cities after the introduction of LEZs [52]; whereas Panteliadis et al. found clear reduction in traffic related air pollutants after the implementation of a LEZ in Amsterdam [50]. The inconsistence of the results could be attributable to the differences in the size of the respective study areas, the duration of the intervention/study period, as well as to differences in how strict the respective entrance requirements were and to differences in the implementation of the respective interventions.

**Greenhouse-gas emission reduction strategies:** Among the 15 articles included, there were two articles with strategies aimed to mitigate climate change or to reach climate change targets [44,48]. The climate mitigation goal could be achieved to some extent either from lower-carbon-emission motor vehicles combined with active travel promotion in this case, or from more stringent climate policies at a global level. Considering that the main air pollutants and greenhouse gases share common sources, air pollution emission reductions, mainly of particulate matter and ozone precursors, were also obtained as a result of these strategies. As for the lower-carbon-emission motor vehicles scenario, PM_2.5_ concentration reduction led to substantial health co-benefits [44]. With a more stringent climate policy at a global level, PM_2.5_ concentrations in the EU would receive an extra cut, and further health benefit would be obtained from both PM_2.5_ and ozone reductions.

### 3.2. Impact on Health and Health Equity

All the strategies from the 15 articles included with simulated or monitored pollutant concentrations demonstrated that the strategies could bring a decrease in ambient air pollution and thus would lead to moderate or substantial health benefit. Beside the general regulations on air quality, specific actions mainly focused on traffic access control (LEZs and CCS), technological innovation to reduce emission (VECs, low-carbon-motor), and energy related emission reduction (improving energy efficiency, energy conservation, or energy switching, e.g., ban of coal sales). Climate change strategies on greenhouse gas emission reductions also reduced air pollutant concentrations and provided related health co-benefit. The results provided mixed but generally suggestive evidence of the effectiveness of air quality control strategies in improving public health, but the effectiveness of those strategies in improving health equity was inconclusive as only three of the articles assessed the SEP impact and the results were not consistent.

For the studies of general regulations on air quality control, the most obvious health benefit was gained through the reduction of particulate matter concentrations. For the studies on energy related strategies, health benefits were obtained mainly from the reduction of SO_2_ and NO_x_ concentrations, and the health benefit covered the entire population of the region covered in the assessment. The health benefit from traffic control interventions (mainly from NO_x_ and particulate matter reductions) was more geographically biased, with a much higher health benefit within the immediate catchment of the interventions. For example, 18.3 YLG per 100,000 population were gained annually in the London Congestion Charge Zone compared with 1.8 YLG in non-Congestion Charge Zones according to Tonne et al. [40]. Furthermore, studies analysing the effects of CCS or LEZs identified a higher reduction of NO_x_ concentrations and subsequent health benefits as compared to particulate matter (annually 18.3 YLG per 100,000 for NO_2_ reduction compared with 6.3 YLG for PM_10_ reduction in the London CCS study; and 45.7 YLG for NO_2_ reduction compared with 7.7 YLG for PM_2.5_ reduction in the Rome LEZs study). The two CCS studies in London and Stockholm showed similar health benefit with 18.3 and 20.6 YLG per 100,000 population gained annually for NO_2_ and NOx concentration reductions respectively. While for LEZs in Rome, the pollutant reductions and health benefits were significantly higher, with 45.7 YLG per 100,000 gained annually for NO_2_ reduction and 7.7 YLG for PM_2.5_ reduction.

Among the articles included, only three articles analysed the impact of the strategies on health equity from socioeconomic position (SEP) perspective. Two articles discussing SEP equity were about traffic control interventions, which were the Congestion Charging Scheme in London and the Limited Traffic Zone in Rome [40,46], and one was about the impact on health equity through meeting UK National Air Quality Strategy targets in Westminster [39]. However, the health equity impacts of these three studies were inconsistent. For the study in London, the research found modest reduction in socioeconomic inequities associated with exposure to traffic related pollution after the introduction of the Congestion Charging Scheme in 2003 [40]. Similarly, as discussed by Mindell and Joffe’s discussed, reducing air pollution could decrease inequities because exposure was likely to be reduced most in socioeconomically deprived areas and because those who benefit most were those with pre-existing health conditions, the very young and older people [39]. On the other hand, the study in Rome by Cesaroni et al. (2011) indicated that most of the health gains were found in well-off residents after the introduction of the Limited Traffic Zone action, hence potentially exacerbating social inequities caused by traffic related air pollutants [46].

Apart from the three studies with SEP assessment, we analysed the effectiveness of the strategies to reduce health inequity using the evidences provided by the reviewed studies. This evidence included differences in the health benefit among socioeconomic groups, age groups, gender groups, pre-existing health condition groups, or geographical groups. In total, among the 12 studies without health equity assessment (Table 3), nine studies mentioned that there were varied health gains either because of the geographical variability [33,37,38,41,43,45], which can be attributable to SEP, or because of the different susceptibility among subgroups [35,36], or both [47]. Subgroups of more susceptible individuals, such as the elderly, children, pregnant women or groups with pre-existing health conditions, were often affected disproportionally. Because the relative risk and the baseline mortality rate were higher for susceptible groups than for the general population, for the same amount of pollutant exposure reduction, susceptible groups were likely to benefit more, and subsequently, this could improve health equity.

## 4. Discussion

This review illustrated that health benefit from air pollution reductions can be gained through all kinds of strategies, actions or plans examined, either as the main goal or as a co-benefit. Because the health benefit was evaluated using different health indicators, the associated pollutants were not always the same, and the exposure–effect terms were often inconsistent (referring to short and/or long term exposure), it was impossible to have a synthesized quantitative evaluation.

Most studies (14) used mortality as the health indicator in the form of avoidable deaths, premature deaths, reduced excess deaths, DALY, YLG, and YLL. Five studies used morbidity in terms of hospitalization or symptoms. Of the 15 studies, four studies monetized health benefits through the willingness to pay (WTP) method using the estimate VOLY obtained from previous studies [33,35,38,42,48]. The methods used (12 out of 15 studies) for health impact assessment involved concentration-response functions (CRF) or exposure-response functions/coefficients (ERF/ERC) obtained either from a series of epidemiologic studies or meta-analysis [2,29,53,54,55,56]. Besides that, one study used a questionnaire survey to obtain the frequency of symptoms associated with air pollutants to assess the health impact of the strategy [37], one study used standardised death rate [36], and one study directly calculated the health gain using the attributable estimate from the WHO health report [47]. Though different among specific groups, all of the examined studies using CRF were based on the assumption that the air pollutant concentrations and health outcomes were linearly related, which might not always be the case [57,58]. Furthermore, for the assessment of the health effects associated with reductions in ozone concentrations, Hutchinson et al. (2004) did not use a threshold (0 ppb), while Schucht et al. (2015) used SOMO35 (ozone concentrations accumulated dose over a threshold of 35 ppb) as a threshold [38,48], indicating some uncertainty in the concentration-response relationship between exposure to ozone and health outcomes. Although most of the health impact assessments considered the local baseline rate of mortality or morbidity (10 articles), or the population change over time (two articles), only two articles controlled for time trends, influenza and temperature effects [33,36]. The only two articles accounting for the geographical variations of population, mortality, and socioeconomic factors included a SEP inequity assessment [40,43]. There were other uncertainties regarding the methodologies used for health impact assessment summarized in this review. Firstly, the air pollution mixture of co-pollutants may differ between the included study areas and the study areas where CRFs/ERFs were obtained; secondly, population-specific time-activity characteristics might differ regionally; and thirdly, the CRFs/ERFs could be different for different social strata, age groups, genders or people with pre-existing health conditions.

The focus of the selected studies was on particulate matter, nitrogen oxides, and sulphur dioxide for some regions in Europe. Particulate matter was the major outdoor air pollutant examined in the general regulation studies, while most strategies in the energy or transport sector result in proportionally larger reductions in NO_x_ and SO_x_ concentrations (Table 3). For example, 2212 premature deaths were estimated to be postponed annually in 20 EU cities because of the SO_2_ reduction through the EU Directives to reduce the sulphur content in liquid fuels for vehicles (EC Directive 93/12/EEC, EC Directive 98/70/EC, and Council Directive 99/32/EC) [33], and 18.3 YLG per 100,000 population were gained annually due to NO_2_ concentration reduction attributed to the Congestion Charge Scheme in London compared to 6.3 YLG per 100,000 for PM_10_ concentration reduction obtained from the same scheme [43]. A large number of the studies only assessed the health impact of one, or few pollutants because of the lack of published concentration-response coefficients [33,38]. As for the interventions normally decrease not merely one pollutant, and because of the interactions between the pollutants, a comprehensive assessment of health benefit of multiple pollutant reductions taking into account co-benefits, would be more appropriate for effectively assessing the health impact of the strategies [59]. Apart from this, other unintended consequences associated with the interventions could also influence health [44]. For example, active travel (e.g., more walking and cycling) could decrease traffic emissions and improve physical activity levels, but potentially increase injury risks, or could increase the exposure time to traffic related air pollutants depending on travel routes [44,60]. As most of the included studies were based on model simulations rather than observations to explore the emission reduction or concentration reduction, results on to what extent the strategies contribute to the pollution reduction and health improvements should be interpreted with caution [59].

Only three studies assessed the impact on SEP health equity, with inconsistent result, indicating a lack of studies on health equity assessment of air quality control strategies. Although we provided comments on the potential impact of the examined strategies on health equity, we cannot draw firm conclusions on which one can decrease or increase health equity, considering specific risks among the varied social gradient. Regarding different population groups, with the same amount of decrease in air pollution concentrations, vulnerable groups were expected to benefit more [35,36,45]. It should also be noted that specific actions (study type II) focusing on typically high-polluted urban areas showed potential to bring about larger health benefits within the geographic catchment area affected by the actions [37,38,43,47]. In summary, air quality control strategies can address air pollution related health inequity by targeting two major pathways: the uneven distribution of concentration of pollutants at various geospatial levels, and the different susceptibilities among population groups. Embedding these two factors into air quality control strategies is advisable for improving the assessment of health equity.

## 5. Conclusions

We conducted a systematic review to identify the effectiveness of the recent strategies relating to air pollution control on public health and health equity in Europe. Fifteen studies were included and four major conclusions can be drawn. Firstly, four groups of strategy type were identified, including general regulations on air quality control, road traffic related emission control interventions, energy generation related emission control interventions and greenhouse gas emission control interventions for climate change mitigation. Secondly, all of these strategies brought improvements in air quality and subsequently in public health either as a direct, intended outcome or as a co-benefit. Only three articles assessed the impact of the strategies on the health equity and the results were inconsistent. Thirdly, the reduction of the air pollutant concentrations and the reported subsequent health benefits were more significant within the geographic catchment of the related interventions. Fourthly, particulate matter (particularly fine particulate matter) and NO_2_ were the main public health concerns related to ambient air pollution in the studies reviewed.

This review not only highlighted the effectiveness and the need for environmental strategies to improve air quality and health, but also explored the connections between socioeconomic status, vulnerability and air pollution exposure. The health co-benefits obtained from the four groups of air pollution control strategies indicated that there was a strong case for promoting Health in All Policies (HiAP) (Table 2), which WHO is facilitating [61], enabling thus possible health improvement from all perspectives. A previous study reviewed air quality control interventions on equity at urban level [62]. To our knowledge, this is the first systematic review of the impact of air quality control strategies on health and health equity in Europe.

This study can contribute to advancing the knowledge related to policies aiming to reduce health risks and health inequity associated with air pollution in Europe. Few limitations still remain. Firstly, language restriction has excluded several national publications from EU member countries; secondly, the search datasets were limited to PubMed, Web of Sciences and TRoPHI; and thirdly, grey literature was excluded.

## Figures and Tables

**Table 1:** Selection criteria for study inclusion and exclusion.

Inclusion	Exclusion
English language for the full article Scientific peer-reviewed articles, including conference articles Europe Union member countries Published between 1 January 1995 till 4 October 2015 Health outcome changes are associated with the concentration change of assessed air pollutants Papers with health assessment from air pollution, indicated by quantitative health indicators, such as mortality, life expectancy, hospital admissions, disease incidence or prevalence, or monetary health benefit, or self-reported health perception, or other indicators which can show the health status Papers focusing on ambient air pollution	Non-English, even with English abstract Government report, project report, etc. Theoretical papers on policies or interventions and related health risks from air pollution Papers that only mentioned health in the conclusions or recommendations Papers assessing interventions that change air pollutant concentrations but not through reduction of emissions from pollution sources (such as green barriers, photocatalytic paints, ventilation, filtration system, etc.) Papers on indoor air pollution control interventions

**Table 2:** Summary of the included studies.

Study & Publish Time	Country & Geographical Scale	Time Period Covered	Strategy or Intervention Description and Study Type (I,II,III)	Methods for Measuring Air Pollution Concentration and Health Outcome, and Brief Study Description	Assessed Air Pollutants ^a^	Health Variables	If Co-Benefit ^b^, Assessment Term, and Cofounders	Target Group
Aunan, K. et al. 1998 [35]	Hungary National level Urban	1992–1993 to the following 5 years	The energy saving program, from National Energy Efficiency Improvement and Energy conservation Programs. (Energy savings of 64 PJ/year ^c^ were expected in a 5 year target period since 1994) **II**	Monitored	NO_2_, SO_2_, TSP ^d^, Dust fallout, PM_10_,	Reduced air pollution attributed annual excess death for >65 and ≤65 years; Reduced air pollution attributed annual excess infant death (0–1 year); Reduced annual acute respiratory symptom days for children and adults; Reduced non-accidental and non-violent mortality; Reduced annual lung cancer cases; Monetary health benefit	Co-benefit Long-term Frequency baseline of the health outcomes	All population, stratified by age group
				Population/recipient data				
				The study simulated the possible reduced damage to public health and other benefits obtained from reducing emissions of key air pollutants				
Clancy, L. et al. 2002 [36]	Ireland Dublin, city level Urban	1984–1990 1990–1996	Ban of coal sales. (The Irish Government banned the marketing, sale, and distribution of bituminous coal within the city of Dublin from 1 September 1990) **II**	Monitored	Black smoke, SO_2_	Annual total non-trauma death; Respiratory death; Car-cerebrovascular death; Other non-trauma death(total minus cardiovascular and respiratory)	Short-term Temperature, relative humidity, day of week, epidemic, standardised cause specific death rate, and age groups	All population, stratified by age group
				Population-standardised death rates				
				The study compared the air pollution concentrations and health before and after the ban of coal sales in Dublin (1990)				
Burr, M.L. et al. 2004 [37]	UK North Wales, district level Urban	Intermittent 1996–2000	By-pass construction in congested area. (A by-pass was opened in an area with severely congested traffic) **II**	Monitored	PM_10_, PM_2.5_	Frequency of symptoms, including wheeze, winter cough, phlegm, consulted doctor, and rhinitis, and peak expiratory flow rate	Short-term Symptom frequency baseline before the intervention	All population, in the experimental area
				Respiratory survey for health				
				The study compared the air pollution concentrations and health outcomes (indicated by the prevalence of respiratory symptoms) between a congested street with a by-pass and uncongested street area				
Hutchinson, E.J. et al. 2004 [38]	UK Country level Urban	1993–1998 1998–2005	Vehicle exhausts catalysts (VECs) (UK mandatorily introduced VECs to gasoline fuelled vehicles since 1993) **II**	Simulated	PM_10_, NO_2_, O_3_^d^, VOCs ^d^, CO ^d^	Monetary health value (all-cause mortality and respiratory hospital admission)	Short-term Population change, underlying mortality rate and underlying hospital admission rate	All population
				Calculated from mortality rate and hospital admission rate				
				The study evaluated the environmental and health benefits of the emission reduction from VECs with available data for exposure assessment and projection for ex ante assessment (1998)				
Mindell, J. and Joffe, M. 2004 [39]	UK Westminster, district level Urban	(1996–1998) 2004–2009	UK National Air Quality Strategy Objectives for 2004 and 2009 **I**	Monitored and targeted	PM_10_	Delayed non-traumatic premature death; Emergency hospital admissions and consultations for respiratory diseases, including asthma, COPD, LRTI, and IHD ^e^	Short and long-term Mortality number and hospital admission baseline	All population, stratified by age groups
				Calculated from routine mortality and hospital admission data				
				The study modelled the health impacts of PM_10_ reduction from the current levels (1996–1998) to the UK 2004 and 2009 target levels				
Tonne, C. et al. 2008 [40]	UK London Central, city level Urban	February 2003–February 2007	Congestion Charging Scheme (CCS) (London Mayor introduced CCS in February 2003) **II**	Simulated	NO_2_, PM_10_	All-cause mortality, indicated by YLG	Co-benefit Long-term Baseline mortality rate, geographic distribution of population and deprivation	All population, stratified by socioeconomic position
				Calculated from mortality data				
				The study modelled the air pollutant concentrations before and after the implementation of CCS, and then used exposure-response coefficients to predict the health gain indicated by years of life gained.				
Ballester, F. et al. 2008 [41]	26 EU cities EU level Urban		European Directive, European Parliament, U.S. Environmental Protection Agency and the World Health Organization on PM_2.5_ guideline (25 μg/m^3^, 20 μg/m^3^, 15 μg/m^3^, and 10 μg/m^3^, respectively) **III**	Monitored & calculated	PM_2.5_	Reduction in all-cause premature deaths; Total burden of all-cause mortality	Long-term Baseline mortality rate	30 years and older
				Calculated from the total mortality data				
				The study estimated the mortality reduction if the PM_2.5_ concentration reduced to the targeted levels				
Perez, L. et al. 2009 [42]	Spain Barcelona 57 municipalities Urban	Post 2004	Directive 2008/50/EC and WHO guidelines for PM_10_ (annual mean concentration of 20 μg/m^3^ and 40 μg/m^3^) **III**	Targeted	PM_10_	Monetary health value, indicated by VOLY ^f^ from all-cause mortality, morbidity (chronic bronchitis and asthma related symptoms), and hospital admissions of respiratory and cardiovascular causes	Short and long-term Population and baseline frequency of mortality and morbidity	All population, with infant death
				Calculated				
				The study estimated the avoided mortality and morbidity under the scenarios examined the annual mean PM_10_ concentration decreased to the WHO recommended level or to the European Union regulatory level				
Johansson, C. et al. 2009 [43]	Sweden Stockholm, city level Urban	2003–2007	Congestion tax system (Stockholm Trial) (Vehicles travelling into and out of the charge cordon were charged for every passage during weekdays) **II**	Monitored and simulated	NO_x_, PM_10_	Premature death, indicated by YLG ^f^	Co-benefit Long-term Baseline mortality rate, geographic distribution of population	All population
				Calculated from the mortality rate				
				The study uses a test trial to measure and model the reduction of road use and then to model the reduction of traffic related PM_10_ and NO_x_; and using epidemiological mortality risk from NO_x_, calculates the avoidable premature death				
Woodcock, J. et al. 2009 [44]	UK London, city level Urban	2010–2030	Road transport interventions (Combination of active travel and lower-carbon emission motor vehicles) ^g^ **II**	Simulated	PM_2.5_	Premature deaths from cardio-respiratory diseases and lung cancer in adults and acute respiratory infections for children DALYs ^f^	Co-benefit Short and long term Physical activity and road traffic accidents	All population stratified by age groups
				Simulated				
				The study compared business as usual and with the interventions, and modelled the health benefit from reduction in PM_2.5_ concentration				
Boldo, E. et al. 2011 [45]	Spain National level Urban and rural	2004–2011	Spain pollution control policies (Spain’s National Emissions Inventory, a baseline 2004 scenario and a projected 2011 scenario on a reduction of primary PM_2.5_, due to technological measures targeting the road transport sector, industry, agriculture, and power generation) **III**	Targeted	PM_2.5_	Avoided all-cause mortality	Long-term Population baseline and mortality baseline stratified by age	30–99 years group; 25–74 years group
				Calculated from the all-cause mortality and population data				
				The study assessed the health benefit under the assumption that specific air quality policies were implemented successfully.				
Cesaroni, G. et al. 2011 [46]	Italy Rome, city level Urban	2001–2005	Limited traffic zone (LTZ) (Without policy scenario, optimistic scenario which assumed that all Euro 0 cars were replaced by Euro 4 cars, and pessimistic scenario which assumed that 10% of Euro 0 cars still running, and the rest 90% of Euro 0 were replaced by Euro 1–4 cars) **II**	Simulated	NO_2_, PM_10_	Total mortality, indicated by YLG	Long-term Distance to the intervention, age groups, education levels	People over 30 years old living along high-traffic road, stratified by the distance of 50 m, 50–100 m and 100–150 m, and stratified by SEP
				Simulated				
				The study calculated the pollution concentration according to the traffic data, and used a concentration-response function to assess the health benefit in two LTZs under the three scenarios				
Chanel, O. et al. 2014 [33]	EU 20 EU cities, EU level Urban	Post 2000	Three European Commission Directives to reduce the sulphur content in liquid fuels for vehicles (1994, 1996, 1999/2000) EC Directive 93/12/EEC, EC Directive 98/70/EC, Council Directive 99/32/EC (Aphekom project). **I**	Monitored & simulated	SO_2_	Annual avoided respiratory, cardiovascular and total premature death (non- external); monetary health benefit indicated by VOLY	Short-term Temperature, day of the week, seasonality, time trend and number of death	All population, in 20 cities in EU
				Calculated from the number of deaths				
				The study compared the emission reduction and health gain before and after the intervention				
Cyrys, J. et al. 2014 [47]	German Berlin, city level Urban	Post 2010	Low emission zones (LEZs) since 2010 **II**	Observed & targeted	Black smoke, PM_10_	Annual avoided total death	Long-term No confounder	All population
				Calculated				
				The study analysed the scientific literatures on the effectiveness of LEZs to PM in German cities and then calculated the avoided death attributable to black smoke due to LEZs in Berlin				
Schucht, S. et al. 2015 [48]	EU EU level Urban and rural	2005–2050	EU air pollution legislation and climate policies **I**	Simulated	PM_2.5_, O_3_	Premature death from acute mortality of respiratory hospital admissions (65+ year) and minor restricted activity days (15–64 year); YLL ^f^ from chronic mortality of all ages; Monetary health benefit, indicated by cost of GDP ^h^	Co-benefit Short and long terms The population change	All population, stratified by age groups
				Simulated				
				The study compared the pollution change and health benefit under the scenario only with air pollution legislation and the scenario with both air pollution legislation and climate policies.				

^a^ For assessed pollutants, we only included the pollutants that were used for health impact evaluation (excluding CO_2_). ^b^ Co-benefit was defined as the additional benefit of strategies which was above or beyond the direct aim of the strategies. ^c^ PJ, petajoule. ^d^ TSP, total suspended particles; O_3_, ozone; VOCs, volatile organic compounds; CO, carbon monoxide. ^e^ COPD, chronic obstructive pulmonary disease; LRTI, lower respiratory tract infection; IHD, ischaemic heart disease. ^f^ YLG, years of life gained; VOLY, value of a life year; DALYs, disability adjusted life years; YLL, years of life lost. ^g^ For strategy A, B and A+B, we only included the one with the highest air pollution concentration reduction and health impact. ^h^ GDP, gross domestic product.

**Table 3:** Summary of health impact assessments and comments on health equity according to the type of the strategies.

Strategy Type	Major Air Pollutants	Reference	Pollution Control Outcome or Targeted Level	Health Outcome *	Was Health Equity Assessed? If Not, Comment on Health Equity
*General regulations on air quality control in Europe*	PM_2.5_, PM_10_	Mindell, J. and Joffe, M., 2004 [39]	PM_10_ concentration with 35 permitted exceedances in 2004 and with 7 exceedances for 2009 for 24 h limit of 50 μg/m^3^; PM_10_ annual mean of 20 μg/m^3^	Avoided 2–39 deaths per 100,000 if complying 2009 24 h PM_10_ target; 3.7–9.3 delayed death if complying UK 2009 annual PM_10_ target in Westminster	Yes, reducing air pollution would decrease inequities because exposure would be reduced most in deprived areas and because those who would benefit most were those with worse health, the very young and older people
		Ballester, F. et al., 2008 [41]	Annual PM_2.5_ dropped to 25 μg/m^3^, 20 μg/m^3^, 15 μg/m^3^, and 10 μg/m^3^, respectively	Annual all-cause premature deaths avoided up to 114 (Cracow) per 100,000 if annual PM_2.5_ dropped to 10 μg/m^3^; Averagely 3% of the total mortality burden among 30 years and older can be reduced	No. Comment: Cracow, Athens and Rome had the most pollution, and benefited the most; London, Dublin and Stockholm had less pollution, and benefited less
		Perez, L. et al., 2009 [42]	Annual mean ambient air PM_10_ concentration dropped from 50 μg/m^3^ to 20 μg/m^3^ (WHO) and to 40 μg/m^3^ (EU)	With WHO target, monetized health benefit was 6400 million Euros per year (1600 euro per capita) from mortality and morbidity	No. Comment: with targeted standards. *No sign* of effect on health equity
		Boldo, E. et al., 2011 [45]	An average annual reduction of 0.7 μg/m^3^ in PM_2.5_ concentration	Annually, 6 per 100,000 population of all-cause deaths avoided for over 30-years group and 5 per 100,000 population avoided for the 25–74 years age group	No. Comment: major absolute health benefits were in Spain’s most densely populated cities, such as Madrid, Barcelona, Seville, and relative benefits were the highest in Andalusia and Mediterranean areas
*Energy related strategies*	SO_2_, NO_x_, TSP, Black smoke	Aunan, K. et al., 1998 [35]	SO_2_ concentration dropped by 5.7%, TSP dropped by 9.3%, NO_x_ dropped by 10.1%, nmVOC (non-methane volatile organic compounds) dropped by 10%, and other greenhouse gases dropped	The program reduced air pollution attributed annual excess death by 9% for the whole population, reduced air pollution attributed annual excess infant death (0–1 year) by 11.4%, reduced annual acute respiratory symptom-days for children and adults by 11.2% and 9.8%, and reduced 25 annual lung cancer cases. The monetized health benefit was 1563 million US dollar	No. Comment: for 65+, infant, and those with pre-existing health conditions, the exceeded cases or exposure days were largely decreased, thus benefited more
		Clancy, L. et al., 2002 [36]	Mean Black smoke concentration dropped by 70% and SO_2_ concentration dropped by 33.8%	Adjusted mortality rate decreased by 5.7% for total non-trauma, 10.3% for cardiovascular, 15.5% for respiratory, 7.9% for less than 60 years group, 6.2% for 60–74 years group and 4.5% for over than 75 years group	No. Comment: the Ban greatly decreased mortality, particularly for cardiovascular disease group
		Chanel, O. et al., 2014 [33]	Gradual decline in SO_2_ concentration	Postponed annual 2212 premature deaths for 20 cities after 2000 comparing with pre 1993; Annual monetized health benefit from mortality was 191.6 million Euro	No. Comment: the lowest number of postponed deaths attributable to the regulation was obtained in Bilbao and the highest in Athens
*Traffic emission control related interventions*	NO_x_, PM	Burr, M.L. et al., 2003 [37]	PM_10_ concentration decreased by 23% (8.0 μg /m^3^) in the congested streets and by 29% (3.4 μg /m^3^) in the uncongested; PM_2.5_ decreased by 23.5% in congested streets and 26.6% in uncongested streets	Clear improvement around the congested streets for rhinitis symptoms, but no clear differences for low respiratory symptoms	No. Comment: the people living along the intervention area benefited more since the intervention covered specific congested area
		Hutchinson, E.J. et al., 2004 [38]	NO_2_ concentration dropped by around 20%, PM_10_ dropped by around 10%, O_3_ increased slightly, VOCs dropped by around 30%, and CO dropped by more than 70%	Net health benefit of 510 million Pound to 1998, and 2157 million Pound to 2005 with the combined concentration change of NO_2_, PM_10_ and O_3_	No. Comment: the city people benefit mostly, the rural areas are unlikely to have large health benefit.
		Tonne, C. et al., 2008 [40]	NO_2_ and PM_10_ concentrations dropped moderately	Total 183 YLG per 100,000 for NO_2_ reduction and 63 YLG for PM_10_ reduction per 10 years in CCS area	Yes, more deprived areas had higher air pollution concentration, and these areas also experienced greater air pollution reductions and mortality benefits compared to less deprived areas
		Johansson, C. et al., 2009 [43]	NO_x_ emission dropped by up to 12%, and PM_10_ dropped by up to 7%	Annually 20.6 YLG per 100,000 people for NO_x_ reduction	No. Comment: the people in the city center (inside the charge cordon) will have the largest reduction in exposure
		Cesaroni, G. et al., 2011 [46]	NO_2_ and PM_10_ concentrations decreased by up to 23% and 10% by the policy	921 YLG per 100,000 along busy road for NO_2_ reduction, average 686 YLG per 100,000 from NO_2_, and 116 YLG per 100,000 for PM_10_ within 150 m of the High traffic Road with the intervention during 15 years	Yes, because wealthy people lived in city center in Rome. High socio-economic population gained most of the health benefit, thus it increased the SEP inequity
		Cyrys, J. et al., 2014 [47]	PM_10_ concentration dropped by up to 10%, and diesel particle dropped by 58%	Annually 144 avoided death per million due to diesel particle decrease	No. Comment: the people living in the zone might benefit more, or people who suffered more from traffic air pollutants benefited more
*Greenhouse-gas emission reduction strategies*	PM_2.5_, O_3_	Woodcock, J. et al., 2009 [44]	PM_2.5_ concentration decreased by up to 9.7%	PM_2.5_ concentration reduction avoided 33 related premature deaths and 319 DALYs per million population	No. Comment: *no sign* of effect on health equity
		Schucht, S. et al., 2015 [48]	For population weighted annual average PM_2.5_ concentration, with mere air quality policies, 75% decrease from 2005 to 2050, with extra 68% reduction if combining climate policies; For SOMO35 (ozone concentrations accumulated dose over a threshold of 35 ppb), 1% increase without climate policies, and 86% decrease with climate policies	Adjusted by the EU population change, for chronic PM_2.5_ mortality, air quality control policies would reduce YLL attributable to PM_2.5_ from 4.6 million to 1 million from 2005 to 2050, with further 300,000 reduction if combining with climate policy. For ozone, premature deaths from acute exposure to ozone would increase from 31,000 to 48,000 from 2005 to 2050, while they would decrease to 7000 with climate mitigation policies at a global level. The monetized health damage would reduce from 3% of the EU GDP in 2005 to 0.4% in 2050 merely with air quality control policies, and to 0.1% if combining with climate policies.	No. Comment: the climate strategies in general decrease the air pollutants (PM_2.5_ and ozone). *No sign* of effect on health equity

* Several studies modelled the health outcomes under different scenarios, but here we only include the one with the largest health benefit.
